# Apatinib Degrades PD-L1 and Reconstitutes Colon Cancer Microenvironment via the Regulation of Myoferlin

**DOI:** 10.3390/cancers17030524

**Published:** 2025-02-05

**Authors:** Chunyi Gao, Lu Chen, Lingying Zhao, Yongcheng Su, Miaomiao Ma, Wenqing Zhang, Xiaoting Hong, Li Xiao, Beibei Xu, Tianhui Hu

**Affiliations:** 1 Xiamen Key Laboratory for Tumor Metastasis, Cancer Research Center, School of Medicine, Xiamen University, Xiamen 361102, China; 21620190154571@stu.xmu.edu.cn (C.G.); 24520180155713@xmu.edu.cn (L.C.); 24520210157060@stu.xmu.edu.cn (Y.S.); 24520221154758@stu.xmu.edu.cn (M.M.); wqzhang@xmu.edu.cn (W.Z.); xthong@xmu.edu.cn (X.H.); 2 Key Laboratory of Prevention and Treatment of Cardiovascular and Cerebrovascular Diseases (Ministry of Education), Gannan Medical University, Ganzhou 341000, China; 3 Department of Laboratory Medicine, Shenzhen Children’s Hospital, Shenzhen 518038, China; miley_zly@163.com; 4 Department of Oncology, Zhongshan Hospital of Xiamen University, Xiamen 361004, China; xlshb0826@xmu.edu.cn; 5 CAS Key Laboratory of Quantitative Engineering Biology, Shenzhen Institute of Synthetic Biology, Shenzhen Institute of Advanced Technology, Chinese Academy of Sciences, Shenzhen 518055, China

**Keywords:** myoferlin, PD-L1, VEGFR2, apatinib, colorectal cancer

## Abstract

Background: For most colorectal cancer (CRC) patients, expanding the benefits of immunotherapy, particularly through blocking programmed cell death-1 (PD-1) and its ligand (PD-L1), is crucial, especially in cases with limited response to neoadjuvant therapy. This study investigates the role of Myoferlin (MYOF) as a novel target in CRC immunotherapy. Methods: Human CRC cell lines (RKO, HCT116), normal intestinal epithelial cells (HIEC-6), and the murine CRC cell line MC38 were used to study the effects of apatinib and MYOF in CRC cells. RNA sequencing, the CPTAC and TCGA databases, and other molecular and cellular methods were applied to disclose the mechanisms involved. A series of mouse models were established to assess the effects of apatinib and MYOF knockdown on tumor progression, immune cell infiltration, and immune checkpoint protein response. Results: We found that MYOF is overexpressed in CRC and linked to immune cell infiltration and checkpoint expression. Suppression of MYOF expression significantly inhibited CRC cell proliferation and migration, as well as reduced PD-L1 protein levels. Integrative analysis showed that apatinib modulates MYOF expression via VEGFR2, resulting in decreased PD-L1 expression, increased CD8+ T cell infiltration, and reduced pro-tumor M2 macrophages. Animal experiments further revealed that apatinib treatment or MYOF knockdown enhanced the efficacy of immune checkpoint blockade (ICB) in CRC. Conclusions: These findings highlight novel antitumor mechanisms of MYOF and suggest that combining apatinib with ICB therapy may improve CRC treatment outcomes, offering a promising strategy to enhance immune responses.

## Simple Summary

For most colorectal cancer (CRC) patients, expanding the benefits of immunotherapy is of great importance. In this study, we found that Myoferlin is highly expressed in CRC and closely associated with immune cell infiltration and checkpoint expression. Importantly, the VEGFR2 inhibitor apatinib promotes the VEGFR2-dependent degradation of Myoferlin, which, in turn, downregulates PD-L1 expression. In vivo, knocking down Myoferlin or using apatinib can remodel the tumor microenvironment and enhance sensitivity to immunotherapy. These findings reveal previously unrecognized mechanisms of Myoferlin and provide a rationale for combining apatinib with immunotherapy in treating CRC.

## 1. Introduction

Colorectal cancer (CRC) is the third most common cancer globally, and its incidence is continuously rising in emerging economies [1] The treatment of CRC primarily relies on traditional surgical resection followed by chemotherapy, but most treated patients still experience recurrence and metastasis [2]. Clinicians are constantly exploring new strategies combining adjuvant chemotherapy to improve compliance, reduce toxicity, and enhance metastasis-free survival. Immunotherapy has been the most exciting advancement in cancer treatment in recent years. The emergence of immune checkpoint blockade (ICB) therapy, especially targeting programmed cell death 1 (PD-1) and its ligand PD-L1, has revolutionized tumor treatment, paving the way for the era of immunotherapy [3]. Among CRC patients, microsatellite instability-high (MSI-H) CRC is one of the earliest tumors shown to benefit from ICB, but the response rate of microsatellite-stable (MSS) CRC patients to ICB therapy is low, with side effects [4,5].

Specifically, for patients with dMMR/MSI-H CRC, the KEYNOTE-177 trial established pembrolizumab monotherapy as the standard first-line treatment [5]. In the CheckMate 142 and CheckMate 8HW studies, combining CTLA-4 blockade with PD-1 inhibitors (nivolumab + ipilimumab) significantly enhanced antitumor immune responses, demonstrating improved efficacy with combination immunotherapy [6,7]. In the neoadjuvant setting for resectable dMMR rectal cancer, studies from NICHE to NICHE-2 and NICHE-3 have consistently shown that dual immunotherapy, whether combining PD-1 inhibitors with CTLA-4 inhibitors or Lag3 inhibitors (relatlimab), significantly improves the pathological complete response (pCR) rate [8,9,10]. In contrast, single-agent immunotherapy has shown limited clinical benefit for pMMR/MSS CRC patients [4,11]. A promising strategy involves combining immunotherapy with agents targeting the VEGF/VEGFR, MEK, or STAT3 pathways to enhance tumor immunogenicity and convert immunologically “cold” tumors into “hot” tumors. The REGONIVO, REGOTORI, and REGOMUN studies demonstrated that VEGF/VEGFR inhibition can normalize tumor vasculature, improve oxygenation, and synergize with immunotherapy by enhancing T-cell priming and activation [12,13,14]. The VETUXIRI and AtezoTRIBE trials further explored combination approaches [15,16]. In the AtezoTRIBE trial, the addition of atezolizumab to FOLFOXIRI + bevacizumab improved progression-free survival (PFS) in MSS CRC patients (12.9 months vs. 11.4 months) [16]. Similarly, the VETUXIRI study showed survival benefits for irinotecan + cetuximab + avelumab in RAS wild-type refractory MSS CRC [15]. The CO.26 trial demonstrated that dual immunotherapy with durvalumab + tremelimumab significantly improved overall survival (OS) in MSS CRC patients [17]. Recently, botensilimab + balstilimab has shown promising results in MSS CRC, with an objective response rate (ORR) of 17%, a disease control rate (DCR) of 61%, and manageable safety profiles, despite a 32% incidence of grade 3–4 treatment-related adverse events [18]. Overall, treating CRC remains challenging, requiring new strategies to regulate the expression of immune checkpoints (ICPs) in most CRC patients to achieve effective immunotherapy [19].

Myoferlin (MYOF) is a member of the myofibroblast protein family with multiple C2 domains [20]. MYOF is highly expressed in skeletal muscle, cardiac muscle, and endothelial cells and plays a crucial role in vesicle transport, cell growth and repair, and receptor-dependent endocytosis [21,22,23]. Recent studies have shown that MYOF is overexpressed in various cancers, including CRC [24,25,26], and high MYOF expression in rectal cancer is associated with poor clinical prognosis in patients [27]. In cancer models, MYOF has been demonstrated to participate in proliferation, invasion, and migration of cancer cells through different mechanisms [28]. MYOF promotes angiogenesis in the tumor microenvironment, is involved in the secretion of exosomes by cancer cells, and enhances the reprogramming of energy metabolism [29,30,31]. These findings suggest that MYOF could be a potential target for new therapeutic strategies.

Vascular endothelial growth factor (VEGF) and its receptors (VEGFRs) play a crucial role in the angiogenesis of solid malignancies. Most of VEGF’s pro-angiogenic effects are believed to be mediated through its binding to VEGFR2 [32]. Upon VEGF stimulation, VEGFR2 forms phosphorylated tyrosine residues in intracellular regions, providing binding sites for downstream signaling molecules [33]. The phosphorylation at specific sites creates binding sites for the SH2 domains of various signaling molecules, ultimately affecting endothelial cell proliferation, migration, and increasing vascular permeability [34]. Apatinib is a multi-targeted tyrosine kinase inhibitor (TKI) that can inhibit targets including VEGFR1, VEGFR2, c-RET, c-KIT, and c-SRC [35]. Apatinib was approved in China in 2014 for the clinical treatment of advanced gastric cancer [36]. So far, apatinib has shown good efficacy against various malignant tumors in clinical settings [35,37,38].

PD-L1 is a 33 kDa protein and a type I transmembrane protein belonging to the immunoglobulin (Ig) superfamily [39]. PD-L1 binds to the receptor PD-1 on activated T cells, inhibiting anti-tumor immunity by counteracting T cell activation signals [40]. The expression of PD-L1 in cells is regulated by various factors at the genomic, transcriptomic, post-transcriptional, and post-translational levels [41]. In the genome, the gene encoding CD274 directly drives the expression of PD-L1. In the transcriptome, immune-stimulatory cytokines such as interferon-γ (IFNγ), IL-6, IFNα, IFNβ, and tumor necrosis factor can induce the expression of PD-L1 [42,43]. Post-transcriptionally, PD-L1 is regulated by the stability of PD-L1 mRNA [44]. Post-translational regulation includes the ubiquitination, glycosylation, and phosphorylation of PD-L1 protein [45]. All these factors collectively contribute to the expression of PD-L1 protein.

Our study found that MYOF stabilizes PD-L1 expression in CRC, reducing the efficacy of ICB. Apatinib, by targeting VEGFR2, promotes the ubiquitination of MYOF, leading to decreased PD-L1 expression, thereby preventing cancer evasion.

## 2. Methods

### 2.1. Data Sources

Proteomics data for CRC patients were downloaded from the Clinical Proteomic Tumor Analysis Consortium (CPTAC) (A-TCGA_Colon_VU_Proteome_CDAP.r). Gene expression profiles were obtained from the Cancer Genome Atlas (TCGA) database (https://cancergenome.nih.gov, accessed on 1 February 2023) (A-diff-TCGA.COAD.sampleMap_HiSeq). Transcriptome data were generated by sequencing normal intestinal cells (HIEC-6) and CRC cells (RKO).

### 2.2. Transcriptome Sequencing Analysis

Total RNA was extracted from human normal intestinal epithelial cells (HIEC-6) and colorectal cancer cells (RKO) using the TRIzol reagent (Vazyme, Nanjing, China) according to the manufacturer’s instructions. The purity and concentration of the extracted RNA samples were measured using a NanoDrop spectrophotometer (ThermoFisher, Waltham, MA, USA) to ensure they met the quality requirements for subsequent sequencing. The transcriptome sequencing and preliminary analysis of the RNA samples were outsourced to BGI Genomics (Wuhan, China).

### 2.3. Differential Expression Analysis

First, differential genes were identified from the transcriptome data of CRC cells. Genes with |log2(fold change)| > 1.5 and adjusted *p*-value < 0.05 were considered statistically significant. Next, genes and proteins highly expressed in both the TCGA and CPTAC [46] databases for CRC tissues were identified, resulting in 36 potential targets. Additionally, the TIMER2.0 online database was used to analyze MYOF protein expression across pan-cancers. The Human Protein Atlas (HPA) online database [47] was further utilized to confirm the intensity of MYOF immunohistochemical staining in CRC. Finally, the UALCAN online database [48] was employed to analyze the MYOF mRNA expression levels between CRC and normal intestinal tissues.

### 2.4. Survival Analysis

The survival probability in CRC for groups with high and low MYOF expression was analyzed using the “Kaplan–Meier Plotter” website (http://kmplot.com/analysis, accessed on 5 February 2023). The optimal threshold was defined by the KM Plotter algorithm, and a *p*-value of <0.05 was considered statistically significant.

### 2.5. Gene Alteration Analysis

The mutation sites of MYOF and VEGFR2 in CRC were evaluated using the cBioPortal database (https://www.cbioportal.org/, accessed on 1 March 2023) [49].

### 2.6. Cell Culture

RKO, HCT116, MC38, HIEC-6, and 293T cell lines were obtained from the Cell Bank of the Chinese Academy of Sciences (Shanghai, China). All cell lines were identified by Short Tandem Repeat profiling by the source. RKO, MC38, and 293T cells were cultured in DMEM medium (Gibco, Waltham, MA, USA) supplemented with 10% fetal bovine serum (FBS, Gibco, USA) and 1% penicillin-streptomycin (100 U/mL penicillin and 100 μg/mL streptomycin). HCT116 cells were cultured in McCoy’s 5A medium (Gibco, USA) supplemented with 10% fetal bovine serum (FBS, Gibco, USA) and 1% penicillin-streptomycin (Gibco, USA). All cells were maintained at 37 °C in a humidified atmosphere containing 5% CO_2_.

### 2.7. Western Blot

Protein lysates (15 μg per sample) from cells or tissues were separated via SDS-PAGE and transferred onto PVDF membranes. The membranes were blocked with 5% non-fat milk and probed with the appropriate primary antibodies followed by HRP-conjugated secondary antibodies. Protein expression was finally detected using a Molecular Imager^®^ Gel Doc™ XR System (Bio-Rad, Hercules, CA, USA) with an enhanced chemiluminescence (ECL) system (P10300, New Cell & Molecular Biotech, Hong Kong, China). The antibodies used in the analysis were as follows: anti-MYOF (A15427, 1:1000 dilution), anti-VEGFR2 (A5609, 1:1000 dilution), and anti-DDDDK-Tag (AE005, 1:5000 dilution) all purchased from ABclonal (Wuhan, China). The anti-PD-L1 antibody (13684, 1:1000 dilution) was obtained from Cell Signaling Technology (Danvers, MA, USA). The anti-Tubulin antibody (66240-1-lg, 1:50,000 dilution) was purchased from Proteintech Group (Rosemont, IL, USA).

### 2.8. RNA Extraction and RT-qPCR

Total RNA was extracted from cells using TRIzol (Takara, Dalian, China) and then used for complementary DNA (cDNA) synthesis with the Primescript™ RT Master Mix (Takara, Dalian, China). Real-time PCR was performed for each target sequence using the FastStart Essential DNA Green Master (Roche, Indianapolis, IN, USA). The expression levels of target genes were determined using the 2 -ΔΔCT method, with glyceraldehyde-3-phosphate dehydrogenase (GAPDH) serving as the internal control. The PCR primer sequences used in the detection are as follows:

- PD-L1-F: TCACTTGGTAATTCTGGGAGC.
- PD-L1-R: CTTTGAGTTTGTATCTTGGATGCC.
- MYOF-F: AAAGTCAGCATGTTTGTCCTGG.
- MYOF-R: GCCTGCCGGAAGTAACAAGTT.
- GAPDH-F: AGGTCGGTGTGAACGGATTTG.
- GAPDH-R: TGTAGACCATGTAGTTGAGGTCA.

### 2.9. Cell Viability Assay

Cells were seeded into 96-well plates. At designated time points, 20 μL of MTT solution (5 mg/mL, Sigma-Aldrich, St. Louis, MO, USA) was added to each well and incubated for 4 h. The medium was then discarded, and the formazan crystals were dissolved in 150 μL of DMSO. Absorbance at 490 nm was measured using a microplate reader (Varioskan Flash, Thermo Fisher Scientific, Waltham, MA, USA).

### 2.10. Cell Migration Assay

RKO and HCT116 cells were seeded into 6-well plates. When the cells reached over 90% confluence, a cross-shaped wound was created in the center of each well using a 200 μL pipette tip. Images of the same area were captured at 0 h and 24 h after the wound was made.

### 2.11. Immunoprecipitation (Co-IP)

For Co-IP, cells were lysed using 0.5% NP-40 lysis buffer (buffer composition: 50 mmol/L Tris-HCl (pH 8.0), 150 mmol/L NaCl, 0.02% sodium azide, 100 µg/mL PMSF, 1 µg/mL aprotinin, and 0.5% Triton X-100) supplemented with protease inhibitors. A total of 2 mg of protein from each sample was diluted in 1 mL of IP lysis buffer. The samples were incubated overnight at 4 °C with an anti-Flag antibody and Protein A + G magnetic beads (Beyotime, Shanghai, China) while gently mixing. A mouse polyclonal IgG antibody (Abclonal, Wuhan, China) was used as a control. After three washes with 0.5% NP-40 buffer, the captured proteins were eluted with 30 µL of 1× SDS-PAGE sample buffer and denatured at 95 °C for 8 min. Finally, the bound proteins were detected using Western blot analysis.

### 2.12. MYOF Protein–Protein Interaction Analysis

To evaluate protein–protein interactions (PPIs) and their alterations due to gene deletions in cancer, we utilized ChiPPI [50]. Seventeen highly correlated genes were identified from the ChiPPI database. Subsequently, a correlation analysis was conducted on CRC data from TCGA using cBioPortal, and Pearson correlation coefficients were calculated.

### 2.13. Single-Cell Analysis of MYOF in CRC

The single-cell analysis of MYOF in CRC was conducted using the Tumor Immune Single-Cell Hub (TISCH) website [51] (http://tisch.comp-genomics.org/, accessed on 1 May 2023). MYOF expression data across 17 different single-cell types were presented in a heatmap format. Additionally, the expression levels of MYOF in different cell clusters were analyzed within the CRC_GSE120909 and CRC_GSE166555 datasets.

### 2.14. Cell Transfection

The plasmids pLV3-CMV-CD274 (human)-3×FLAG-Puro (P64013), pLV3-CMV-MYOF (human)-3×FLAG-CopGFP-Puro (P50257), and pLV3-CMV-KDR (human)-3×FLAG-EF1a-CopGFP-Puro (P36517) were obtained from MiaoLingBio (Wuhan, China). For transient transfection, cells were transfected using Lipo6000™ transfection reagent according to the manufacturer’s instructions, and the cells were analyzed 48 h post-transfection. For lentiviral transduction, lentiviruses were produced by transfecting HEK293T cells with plasmids using PEI. The culture medium containing lentivirus was collected 48 h after transfection, and Polybrene (28728-55-4, Sigma, St. Louis, MO, USA) was added. RKO, HCT116, and MC38 cells were infected by replacing their culture medium with the viral supernatant for 8 h. Stable clones were selected using puromycin.

### 2.15. Ubiquitination Assay

Ubiquitination assays were performed under denaturing conditions in this study. Ubiquitination was detected using denaturing immunoprecipitation (d-IP). RKO cells with stable MYOF knockdown and control RKO cells were transfected with HA-ubiquitin and Flag-PD-L1 for 48 h, followed by treatment with 20 µM MG132 for 4 h. Cells were then resuspended in ice-cold 0.5% NP-40 buffer (50 mM Tris-HCl, pH 7.5, 150 mM NaCl, 0.5% NP-40, 100 µg/mL PMSF, 1 µg/mL aprotinin, and 0.5% Triton X-100). Protein concentration was determined using a BCA Protein Assay Kit (Thermo Fisher Scientific). The lysates were denatured by boiling in 2% SDS for 15 min. After adjusting the SDS concentration to 0.5%, the cell lysates were incubated overnight with anti-Flag antibody and magnetic beads. The following day, proteins were eluted from the beads and subjected to immunoblotting.

### 2.16. Animal Experiments

C57BL/6 female mice (4–6 weeks old) were purchased from Shanghai Slack Laboratory Animal Co., Ltd. and housed in SPF-grade facilities at Xiamen University. The mice were kept in individually ventilated cages under a 12-h light-dark cycle at 25 °C and a humidity level of 40–60%. They had free access to food and sterile water. In the animal experiments, MC38 cells with stable MYOF knockdown were generated using lentiviral transfection. C57BL/6 mice were then subcutaneously injected with either MYOF knockdown MC38 cells or control MC38 cells (5 × 10^5^ cells). When the average tumor volume reached approximately 50–100 mm^3^, the mice were randomly divided into groups. The treatment consisted of oral administration of apatinib (200 µL, 60 mg/kg) daily and intraperitoneal injection of anti-PD-1 (200 µg) (BE0146, Biocell, Littleton, MA, USA) three times a week. Tumor volumes were measured every 5 days using a caliper, with the volume calculated using the formula: Volume = (a × (b)^2^)/2, where “a” represents the longest diameter and “b” is the shortest diameter. When the maximum tumor diameter reached 2 cm or if the tumor became necrotic, mice were euthanized by CO_2_ asphyxiation. All animal experiments were approved by the Xiamen University Animal Care and Use Committee (Protocol No. XMULAC20220179).

### 2.17. Enzyme-Linked Immunosorbent Assay (ELISA)

Tumor tissues were homogenized and diluted, according to the manufacturer’s instructions for the ELISA kit (EK0513, BOSTER, Wuhan, China). TGF-β in the homogenate was detected.

### 2.18. Flow Cytometry

After the in vivo experiments, tumors and spleen tissues were harvested from mice. Tissue blocks (1 cm^3^) were ground into single-cell suspensions using a 40 μm filter mesh screen. Cells were counted, and 2 × 10^6^ cells were divided into two portions. For T cell subset analysis, 1 × 10^6^ cells were stained with the recommended amounts of anti-mouse CD45-BV421 (BioLegend, cat. #103134, San Diego, CA, USA), CD3-PE (BioLegend, cat. #100308, CA, USA), CD4-APC (BioLegend, cat. #100412, CA, USA), and CD8a-Percp-cy5.5 (BioLegend, cat. #100734, CA, USA) in PBS and incubated at 4 °C for 30 min. For monocyte subset analysis, the other 1 × 10^6^ cells were stained with anti-mouse F4/80-BV421 (BioLegend, cat. #123132, CA, USA), CD11b-APC (BioLegend, cat. #101262, CA, USA), CD11c-PE (BioLegend, cat. #117308, CA, USA), and I-A/I-E-PE-cy7 (BioLegend, cat. #107630, CA, USA) at 4 °C for 30 min. Cells were then fixed at room temperature, permeabilized with 1% Tween 20, and stained with anti-mouse CD206-BV650 (BioLegend, cat. #141723, CA, USA) at 4 °C for 30 min. Cells were resuspended, filtered, and analyzed using the CytoFLEX S (Beckman, Brea, CA, USA). Data analysis was performed using FlowJo software Version 9.9.5.

### 2.19. Immunohistochemistry (IHC)

After fixation, embedding, and sectioning of tumor tissues, staining was performed using the HRP-Polymer anti-Mouse/Rabbit IHC Kit (KIT-9710/9720/9730, MaxVision, Shenzhen, China) and anti-TGF-β antibody (ab215715, Abcam, Cambridge, UK) according to the manufacturer’s instructions. Images were captured using the Motic VM1 Digital Slide Scanning System (Hong Kong, China). All immunostaining images were analyzed using the IHC Profiler plugin in ImageJ [52] (http://rsb.info.nih.gov/ij/plugins/ihc-toolbox/index.html, accessed on 1 July 2023).

### 2.20. Protein Extraction

For protein extraction, cells in a 6 cm culture dish were lysed with 500 μL of ice-cold RIPA lysis buffer containing PMSF, NaVO3, and β-glycerophosphate to inhibit proteases and phosphatases. After a 10-min incubation on ice, cells were scraped, collected into labeled tubes, and disrupted using ultrasonication (20% amplitude, 12–15 cycles of 2 s each). The lysate was centrifuged at 13,200 rpm for 15–20 min at 4 °C to remove debris. The supernatant was carefully collected, and protein concentration was determined using the BCA method. Samples were diluted with 5× loading buffer, boiled for 8 min for denaturation, and stored for subsequent analysis.

### 2.21. Statistical Analysis

Statistical analysis was performed using GraphPad Prism Version 9 (GraphPad Software, La Jolla, CA, USA). A *p*-value less than 0.05 was considered statistically significant (* *p* < 0.05; ** *p* < 0.01; ns, not significant). Statistical comparisons were made using an unpaired t-test (for comparisons between two different groups) or one-way ANOVA with Tukey’s multiple comparisons test. Correlation coefficients were determined using Pearson correlation analysis.

## 3. Results

### 3.1. The Expression of MYOF Is Higher in CRC

**Figure 1:**
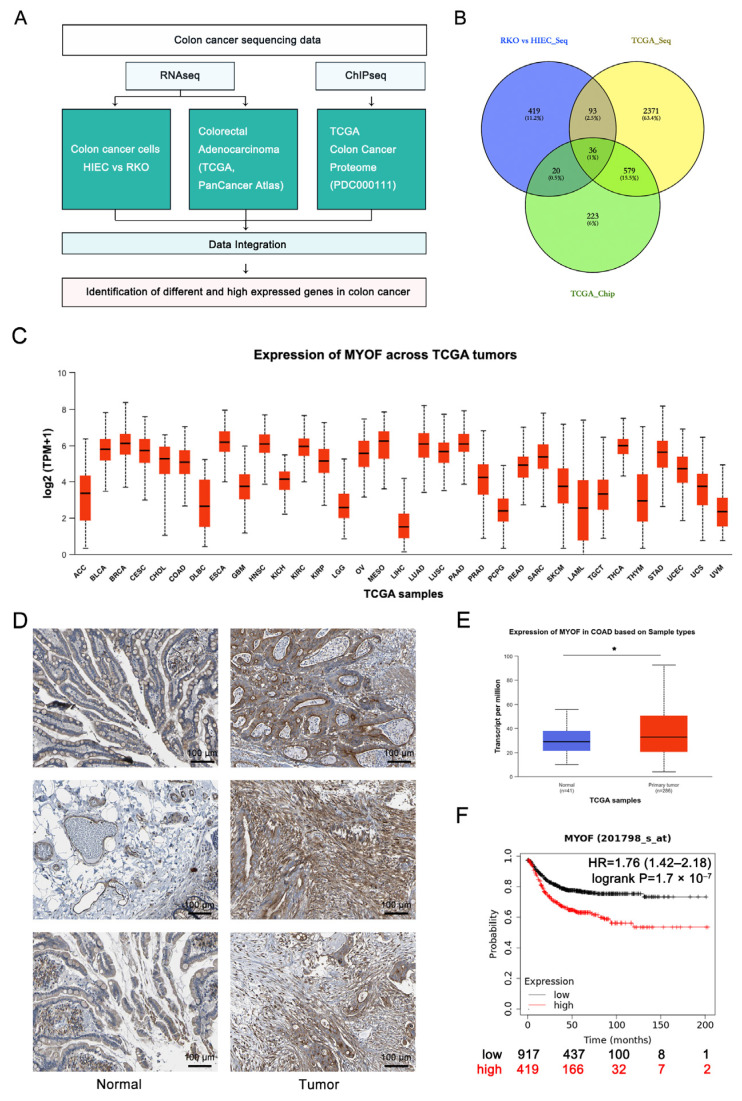
The expression of MYOF is higher in CRC. (**A**) A flowchart showing the process for identifying abnormally activated genes in CRC. RNA sequencing (RNA-seq) was performed on HIEC-6 and RKO cells to analyze differentially expressed genes. Data from 180 CRC patients were collected from the TCGA database, focusing on genes with high expression levels (top 15%). Public proteomics data from 95 tumor samples, including 90 patients, were collected from CPTAC, also focusing on highly expressed genes (top 15%). The data were integrated to identify potential target genes for further research. (**B**) A Venn diagram illustrating the number of target genes identified in each CRC data analysis. Thirty-six target genes were common across the three types of analysis. (**C**) MYOF mRNA expression across various cancers, as shown by the UALCAN website. (**D**) Representative images of MYOF staining in tumor and adjacent non-tumor tissues from CRC patients, obtained from the HPA database. The scale bar represents 100 µm. (**E**) A comparison of MYOF levels in normal and primary CRC tissues from the UALCAN database. (**F**) Overall survival rates of patients with low and high MYOF expression, as analyzed using the Kaplan–Meier database (* *p* < 0.05).

To explore potential elements that activate CRC, we performed RNA sequencing data analysis on normal human intestinal epithelial cells (HIEC-6) and human colon cancer cells (RKO). A total of 3793 genes were identified as significantly differentially expressed, including 2590 genes with significantly elevated expression levels in cancer cells compared to normal cells (Supplementary Figure S1A). Notably, among the upregulated genes, MYOF exhibited a prominent position in the volcano plot generated from the RNA-seq data (Supplementary Figure S1B). Subsequently, we analyzed the sequencing results of CRC tissues from the PanCancer Atlas and collected proteomics data of CRC patients from the CPTAC database. (Figure 1A). We integrated and analyzed the data to identify genes and proteins that are lowly expressed in normal intestinal epithelial cells but abnormally activated in CRC cells and tissues (Figure 1B). Analysis of the TCGA database revealed that MYOF mRNA expression levels are elevated in various human cancer tissues, including CRC (Figure 1C). IHC staining data from the HPA database indicated higher MYOF protein expression in CRC compared to adjacent tissues (Figure 1D). Similarly, the UALCAN website analysis shows that MYOF mRNA is significantly overexpressed in primary colorectal tumors (*n* = 286) compared to normal colorectal tissues (*n* = 41, *p* = 0.0236) (Figure 1E). Using the Kaplan–Meier plotter to compare MYOF expression in CRC, the data indicated that patients with high MYOF expression had lower OS compared to those with low MYOF expression (*p* < 0.0001) (Figure 1F). This suggests that MYOF expression levels may influence survival in CRC, making MYOF a potential prognostic predictor for CRC patients.

### 3.2. MYOF Knockdown Inhibits Proliferation of CRC Cells In Vitro

**Figure 2:**
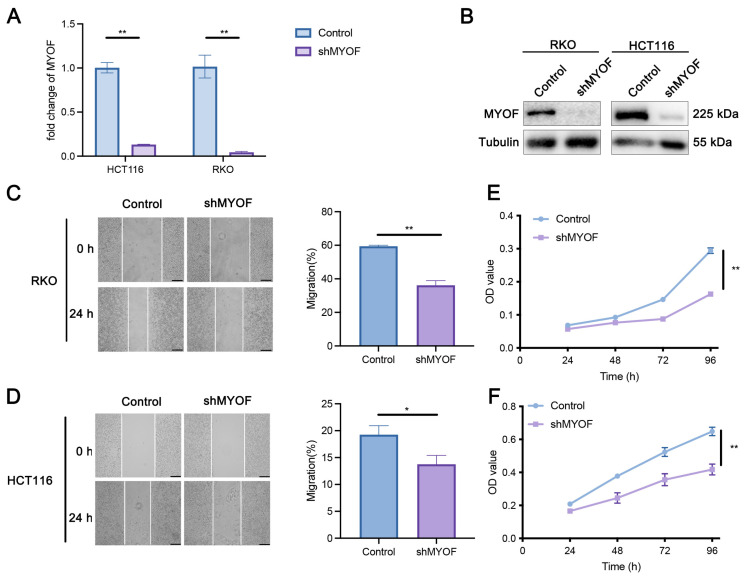
MYOF knockdown significantly inhibits the proliferation and growth of CRC cells in vitro. (**A**,**B**) After CRC cells were infected with MYOF-targeting shRNA virus, MYOF expression was assessed using RT-qPCR and Western blot analysis. (**C**,**D**) Wound healing assays were used to compare the migratory ability of RKO and HCT116 cells with or without MYOF knockdown. Scare bar = 100 μm. (**E**,**F**) MTT assays were used to compare the viability of RKO and HCT116 cells with or without MYOF knockdown (* *p* < 0.05; ** *p* < 0.01). The original images of the Western blotting figures can be found in Supplementary File S1.

The aforementioned bioinformatics results suggest that MYOF may play a significant role in the development and progression of CRC, warranting further experimental validation. Consequently, we conducted in vitro experiments to confirm the critical role of MYOF in CRC cell lines. RKO and HCT116 cells were transfected with MYOF shRNA. RT-qPCR showed a significant decrease in MYOF mRNA, indicating successful inhibition (*p* = 0.0001, *p* = 0.0017) (Figure 2A). Western blotting confirmed reduced MYOF protein in the knockdown group, validating gene silencing (Figure 2B). Wound healing assays demonstrated that MYOF knockdown inhibited the migratory ability of HCT116 and RKO cells (*p* = 0.0082, *p* < 0.0001) (Figure 2C,D). MTT assays revealed that MYOF knockdown exhibited a significant antiproliferative effect on RKO and HCT116 cells (*p* < 0.0001, *p* < 0.0001) (Figure 2E,F). In summary, MYOF is a potential oncogene, and its reduced expression impairs the in vitro proliferative and migrative capacity of CRC cell lines.

### 3.3. Interaction Between MYOF and VEGFR2

**Figure 3:**
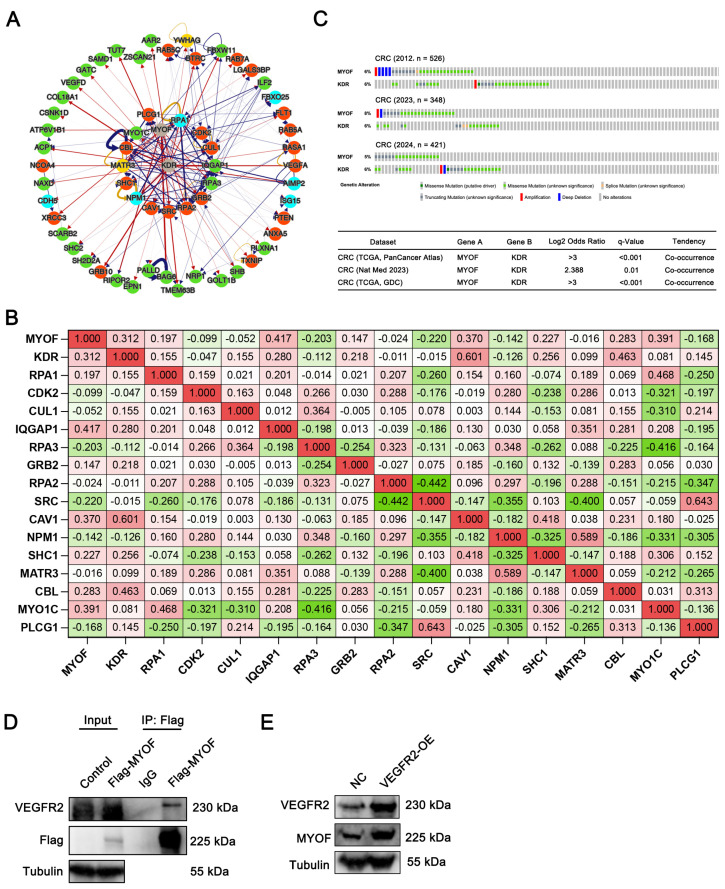
Interaction between MYOF and VEGFR2. (**A**) Protein–protein interaction network analysis reveals the cellular connections between MYOF and VEGFR2, mediated by the Chimeric Protein–Protein Interaction (ChiPPI) method. (**B**) Correlation analysis of MYOF, KDR (VEGFR2), RPA1, RPA2, RPA3, CDK2, CUL1, IQGAP1, GRB2, SRC, CAV1, NPM1, SHC1, MATR3, CBL, MYO1C, and PLCG1 levels in human CRC tissues (TCGA, *n* = 524). (**C**) Data for CRC from the TCGA PanCancer Atlas, Nature Medicine, and GDC datasets were extracted from cBioPortal. Oncoprint provides an overview of the genetic variations and expressions of MYOF and VEGFR2 across three batches of CRC samples. The colors indicate gene alterations, as shown in the legend. In the table, a co-occurrence panel analysis depicts the simultaneous alterations of MYOF and VEGFR2 genes in CRC. *p*-values were calculated using a one-sided Fisher’s exact test and adjusted using the Benjamini–Hochberg correction. (**D**) Co-IP assay in RKO cells stably expressing Flag-MYOF. Immunoprecipitation was performed using anti-IgG or anti-FLAG antibodies, followed by Western blotting for MYOF. (**E**) Western blot analysis for MYOF in RKO cells stably expressing VEGFR2. The original images of the Western blotting figures can be found in Supplementary File S1.

Research has demonstrated that in HUVEC cells, MYOF might interact with VEGFR2 and dynamin-2 by forming a complex via its SH3 domain [53]. We conducted a network analysis to better understand the interaction between MYOF and VEGFR2. Specifically, we utilized a chimeric protein–protein interaction method (ChiPPI) to predict alterations in the cellular network. The resulting network comprised 17 nodes: MYOF, KDR (VEGFR2), RPA1, RPA2, RPA3, CDK2, CUL1, IQGAP1, GRB2, SRC, CAV1, NPM1, SHC1, MATR3, CBL, MYO1C, and PLCG1 (Figure 3A). Using the TCGA database, we further analyzed the correlations between the mRNA expressions of these nodes. Our findings revealed that MYOF positively correlated with KDR (VEGFR2) (R = 0.312, *p* < 0.05) (Figure 3B). Using the Oncoprint tool available on the cBioPortal website, we examined the mutation frequencies of MYOF and VEGFR2 across three CRC datasets. Mutual exclusivity analysis of these data consistently demonstrated a significant co-occurrence trend between MYOF and VEGFR2 (Figure 3C). The aforementioned bioinformatics results suggest that MYOF may play a crucial role in the progression of CRC and is potentially linked with VEGFR2. These findings warrant further experimental validation. To this end, a Co-IP assay was performed in the CRC cell line RKO, and the results confirmed the interaction between MYOF and VEGFR2 (Figure 3D). To determine whether VEGFR2 can regulate MYOF expression in CRC cells, we manipulated the expression of VEGFR2. As illustrated in Figure 3E, MYOF expression increased concomitantly with the upregulation of VEGFR2. These data suggest that MYOF and VEGFR2 interact with each other.

### 3.4. Apatinib Promotes MYOF Protein Degradation

**Figure 4:**
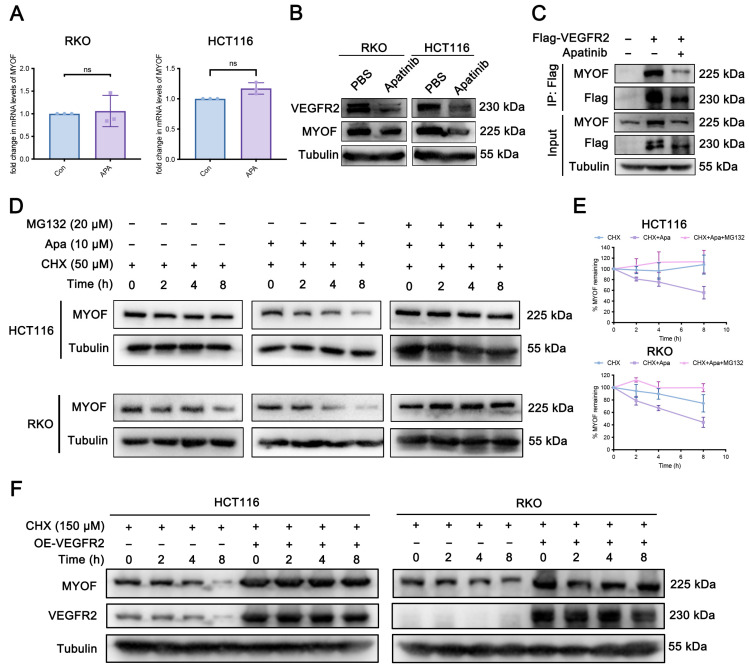
Apatinib promotes MYOF protein degradation. (**A**) MYOF mRNA expression in RKO and HCT116 cells treated with 20 μM apatinib for 48 h. (**B**) MYOF protein expression in RKO and HCT116 cells treated with 20 μM apatinib for 48 h. (**C**) RKO cells stably expressing Flag-MYOF were treated with apatinib or PBS for 24 h. Immunoprecipitated samples (upper panel) and input samples (lower panel) were analyzed by Western blotting for MYOF and FLAG. (**D**) MYOF protein expression in RKO and HCT116 cells treated with CHX alone, CHX combined with 10 μM apatinib, or CHX combined with apatinib and MG-132 (20 μM) at different time points. (**E**) Quantification of MYOF protein levels and plotting of protein half-life in RKO and HCT116 cells under the indicated treatments. (**F**) Western blot analysis of MYOF protein expression in RKO and HCT116 cells co-transfected with Flag-VEGFR2 and treated with 150 μM CHX for the indicated time intervals. ns, not significant. The original images of the Western blotting figures can be found in Supplementary File S1.

The above results indicate that MYOF might be regulated by VEGFR2. As a VEGFR2 inhibitor, apatinib might also regulate MYOF through inhibition of VEGFR2 [54]. To further study the effects of apatinib on MYOF, we measured the mRNA and protein levels of MYOF in CRC cells treated with apatinib. Treatment with apatinib in RKO and HCT116 cells did not result in significant changes in MYOF mRNA levels (*p* = 0.4274, *p* = 0.1690) (Figure 4A), but it did reduce protein expression (Figure 4B). Apatinib reversed the VEGFR2 overexpression-induced upregulation of MYOF expression, and the interaction between MYOF and VEGFR2 was reduced upon apatinib treatment (Figure 4C). Further cycloheximide (CHX) chase assays demonstrated that apatinib significantly enhanced MYOF protein degradation, and the proteasome inhibitor MG-132 markedly altered MYOF expression levels in apatinib-treated CRC cells, suggesting that MYOF protein may be degraded through the ubiquitin–proteasome system (Figure 4D,E). To further elucidate if MYOF stability is related to VEGFR2, we overexpressed VEGFR2 and the result showed that VEGFR2 increased MYOF stability (Figure 4F). These data suggest that apatinib acts as a negative regulator of MYOF by promoting its degradation through a VEGFR2-related proteasome pathway (* *p* < 0.05; ** *p* < 0.01; ns, not significant).

### 3.5. MYOF Associates with the Tumor Immune Microenvironment

**Figure 5:**
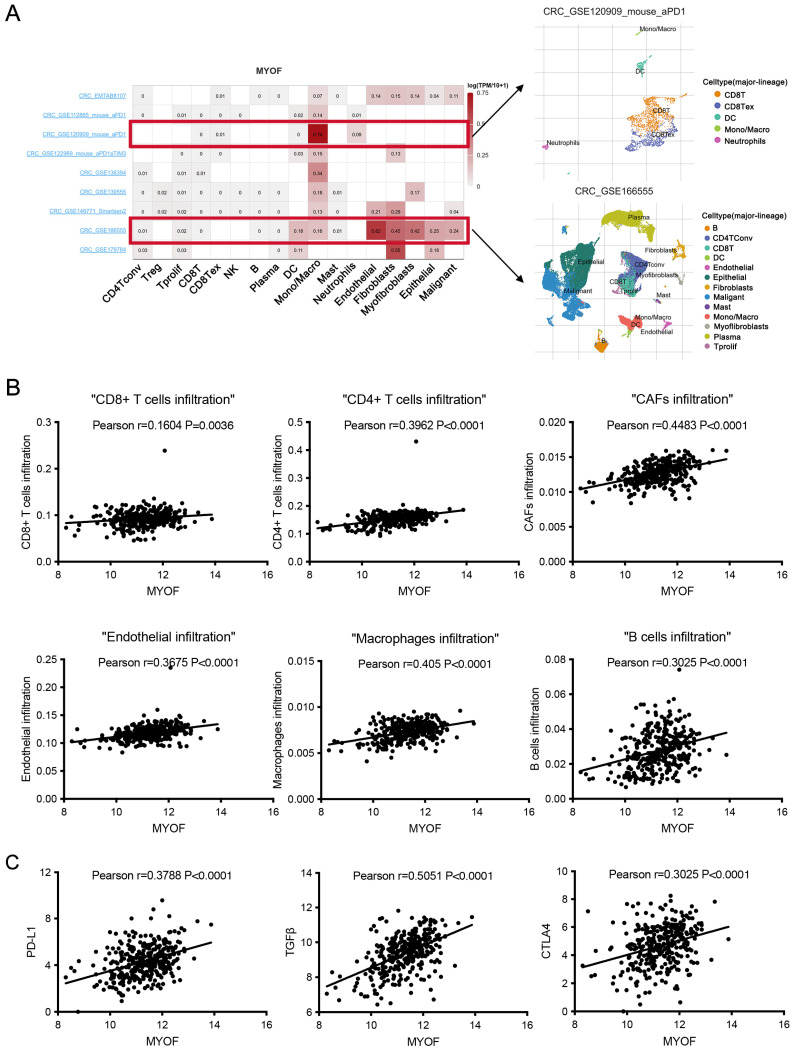
Immuno-correlation analysis of MYOF. (**A**) Single-cell expression dataset of MYOF in nine CRC samples from the TISCH website. (**B**) Correlation between MYOF expression and immune infiltration levels of T cells, CAFs, endothelial cells, macrophages, and B cells. (**C**) Correlation between MYOF expression and immune checkpoints PD-L1, TGF-β, and CTLA-4. Spearman correlation analysis was performed.

The tumor microenvironment consists of tumor cells, stromal cells, immune cells, and various small molecular factors secreted by these cells, such as growth factors and chemokines. These components work together within the microenvironment, influencing the occurrence and development of CRC. To understand the primary cellular distribution of MYOF expression in tumor tissues, single-cell sequencing data from nine CRC datasets in the TISCH2 database were analyzed. The results show that MYOF is expressed not only in malignant tumor cells but also widely across most types of immune cells. Using the CRC_GSE120909 dataset (PMID: 30389797), the gene expression of 1881 immune cells in mice was analyzed, revealing that MYOF is broadly expressed in monocytes/macrophages. In the CRC_GSE166555 dataset (PMID: 34409732), the gene expression of 66,050 immune cells from 12 patients was analyzed, showing that MYOF is highly expressed in endothelial cells, fibroblasts, and myofibroblasts (Figure 5A). Spearman’s rank correlation (Spearman’s r) and ssGSEA were used to analyze the correlation between MYOF and immune cell subsets in CRC. We found a high correlation between MYOF in CRC and CD4+ T cells (R = 0.3962, *p* < 0.0001), CAFs (R = 0.4483, *p* < 0.0001), endothelial cells (R = 0.3675, *p* < 0.0001), macrophages (R = 0.405, *p* < 0.0001), and B cells (R = 0.3025, *p* < 0.0001) (Figure 5B). Furthermore, we investigated the relationship between MYOF expression and ICP genes in CRC to explore the potential of MYOF in immunotherapy. The results show that MYOF expression is highly positively correlated with PD-L1 (R = 0.3788, *p* < 0.0001), TGF-β (R = 0.5051, *p* < 0.0001), and CTLA-4 (R = 0.3025, *p* < 0.0001) (Figure 5C). In summary, our findings suggest that MYOF may remodel an immunosuppressive TME by upregulating the expression of ICPs.

### 3.6. Apatinib Promotes PD-L1 Ubiquitination and Reduces PD-L1 Expression via MYOF

**Figure 6:**
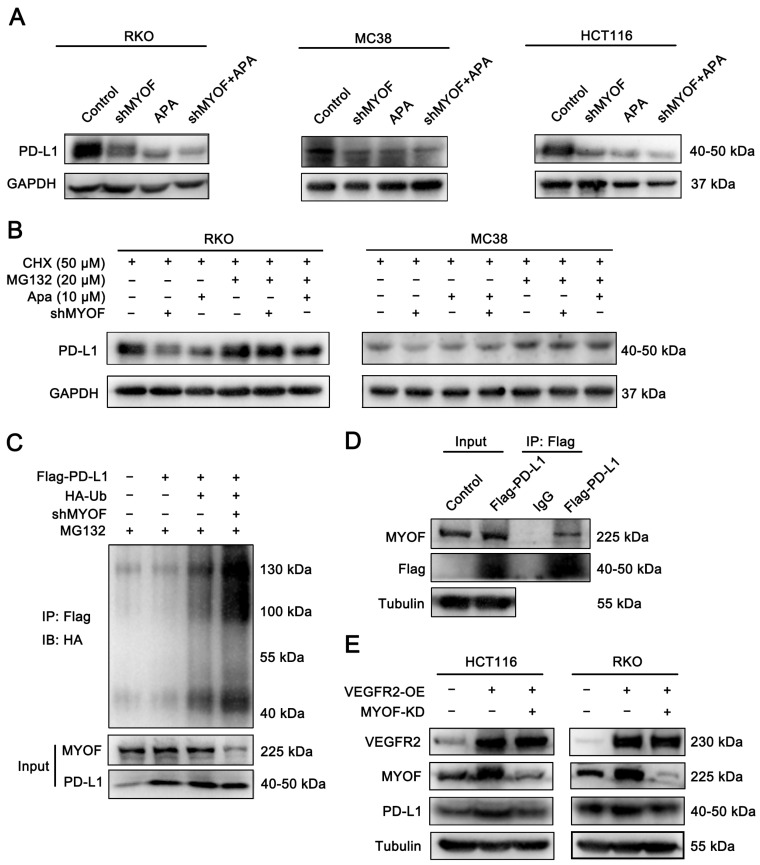
Apatinib promotes PD-L1 ubiquitination and reduces PD-L1 expression via MYOF. (**A**) CRC cells were infected with shRNA viruses targeting MYOF, and PD-L1 expression in RKO, MC38, and HCT116 cells was analyzed by Western blotting, comparing conditions with and without apatinib treatment (20 µM, 48 h) or MYOF knockdown. (**B**) PD-L1 protein levels were measured in RKO and MC38 cells after treatment with MG132 (20 µM) for 6 h, following apatinib treatment (20 µM, 48 h) or MYOF knockdown. (**C**) Polyubiquitination of PD-L1 was assessed by immunoprecipitation in RKO cells with PD-L1 overexpression or MYOF knockdown. Cells were treated with MG132 (10 µM) for 4 h before harvesting. (**D**) A co-immunoprecipitation (Co-IP) assay was performed in RKO cells stably expressing Flag-PD-L1. Immunoprecipitation was conducted using anti-IgG or anti-FLAG antibodies, followed by Western blotting for MYOF. (**E**) PD-L1 expression was analyzed by Western blotting in RKO and HCT116 cells with VEGFR2 overexpression or MYOF knockdown. The original images of the Western blotting figures can be found in Supplementary File S1.

The aforementioned immunoinformatic analysis suggests that MYOF may act as a regulator of PD-L1. Previous work by our research group has demonstrated a decrease in surface PD-L1 expression on CRC cells following apatinib treatment [55]. These findings collectively present an intriguing case: whether apatinib regulates PD-L1 expression through MYOF. To elucidate the specific mechanism by which apatinib modulates PD-L1, we assessed PD-L1 mRNA and protein levels in CRC cells with MYOF knockdown or after apatinib treatment. Neither MYOF knockdown nor apatinib treatment reduced PD-L1 transcription levels (Supplementary Figure S2). However, the protein levels were significantly decreased in RKO, HCT116, and MC38 cell lines (Figure 6A). Subsequently, MYOF knockdown or apatinib-treated cells with the proteasome inhibitor MG132 led to a marked restoration of PD-L1 protein levels in RKO and MC38 cells (Figure 6B). Further ubiquitination assays revealed that MYOF knockdown promoted PD-L1 ubiquitination in RKO cells (Figure 6C), suggesting that MYOF might influence the stability of PD-L1, with potential interactions between MYOF and PD-L1 proteins. Introducing flag-PD-L1 expression plasmid into RKO cells and performing Co-IP confirmed the binding between MYOF and PD-L1 proteins (Figure 6D). We have previously demonstrated an interaction between VEGFR2 and MYOF (Figure 2). Consistently, VEGFR2 overexpression resulted in increased PD-L1 protein levels in HCT116 and RKO cells, while MYOF knockdown reversed the elevated PD-L1 expression (Figure 6E). These results indicate that apatinib facilitates MYOF degradation via VEGFR2, thereby regulating PD-L1 ubiquitination and proteasomal degradation in CRC.

### 3.7. Apatinib Treatment or MYOF Knockdown Significantly Inhibits Colon Cancer Tumor Growth and Remodels the Tumor Immune Microenvironment

**Figure 7:**
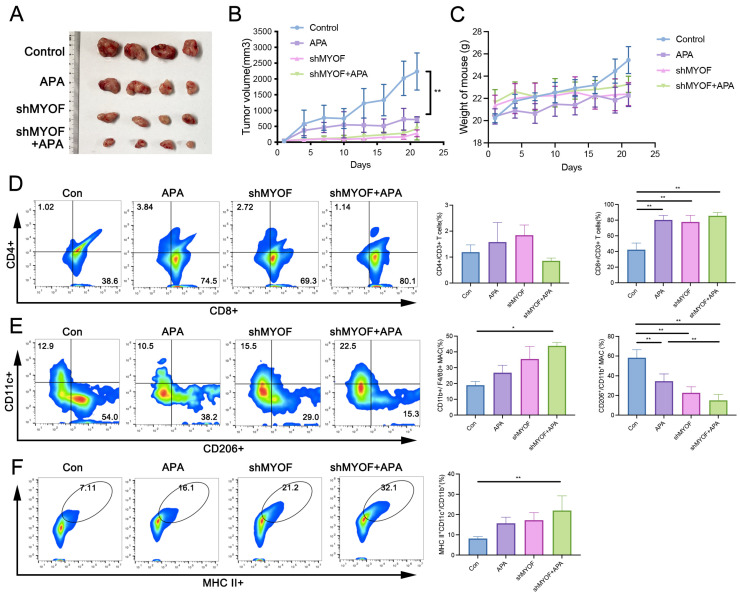
Apatinib treatment or MYOF knockdown significantly inhibits colon cancer tumor growth and remodels the tumor immune microenvironment. (**A**) Representative images of tumors in immunocompetent C57BL/6 mice (*n* = 4) treated with apatinib gavage, MYOF knockdown, and the combination regimen using MC38 or MC38 shMYOF cells. (**B**) Tumor growth curves in mice across different treatment groups. (**C**) Body weight curves of mice in each group. (**D**) FACS analysis of CD8+ and CD4+ tumor-infiltrating lymphocytes. (**E**) FACS analysis of tumor-infiltrating M1 (CD11c+ CD11b+) and M2 (CD206+ CD11b+) macrophages. (**F**) FACS analysis of activated DCs (MHCii) in tumor tissues. (* *p* < 0.05; ** *p* < 0.01).

Next, we sought to determine whether apatinib or MYOF knockdown could restrict tumor growth in vivo using an immunocompetent MC38 mouse cancer model. We subcutaneously implanted MC38 shMYOF cells into C57BL/6 mice and found that both MYOF knockdown and apatinib gavage treatment significantly inhibited tumor growth, leading to tumor shrinkage. The combination of MYOF knockdown and apatinib treatment had a comparable effect to either treatment alone (Con vs. APA, *p* = 0.0003; Con vs. shMYOF, *p* < 0.0001; Con vs. shMYOF + APA, *p* < 0.0001) (Figure 7A,B). No significant differences in body weight were observed during the administration period (Figure 7C). This suggests that apatinib and MYOF knockdown may inhibit tumor progression through the same pathway. To further elucidate the impact of MYOF knockdown on the tumor microenvironment, we analyzed tumor-infiltrating lymphocytes (TILs) in MYOF-KD tumors in mice. Our findings indicate a significant increase in CD8+ T cell infiltration across treatment groups, including apatinib alone, MYOF knockdown, and their combination, suggesting an enhanced cytotoxic T cell activity. Conversely, changes in CD4+ T cell infiltration were insignificant, pointing to a lesser impact on helper T cells (Con vs. APA, *p* < 0.0001; Con vs. shMYOF, *p* < 0.0001; Con vs. shMYOF + APA, *p* < 0.0001) (Figure 7D). Further analysis revealed an overall increase in macrophage levels in the combination treatment group, likely linked to an enhanced inflammatory response within the tumor microenvironment (*p* = 0.0354). The proportion of pro-tumorigenic M2 macrophages was significantly reduced in all treatment groups, which could alleviate immune suppression and promote a more effective antitumor immune response (Con vs. APA, *p* = 0.0021; Con vs. shMYOF, *p* < 0.0001; Con vs. shMYOF + APA, *p* < 0.0001; APA vs. shMYOF + APA, *p* = 0.0089) (Figure 7E). Furthermore, an increase in the proportion of activated dendritic cells was observed in mice treated with MYOF knockdown and apatinib. Dendritic cells, as principal antigen-presenting cells, play a pivotal role in initiating and regulating T cell responses. The enhanced activity of dendritic cells may facilitate improved presentation and processing of tumor antigens, further activating T cell-mediated immune responses (*p* = 0.0036) (Figure 7F). Additionally, we assessed the infiltration levels of CD4+ and CD8+ T cells in the spleen and found no significant differences between the groups (Supplementary Figure S3). This indicates that apatinib treatment or MYOF knockdown primarily influences the changes in immune cell infiltration within the tumor rather than in the spleen.

Transforming Growth Factor-β (TGF-β) is a multifunctional cytokine. TGF-β signaling is responsible for orchestrating the immunosuppressive TME and supports cancer growth, invasion, metastasis, recurrence, and treatment resistance [56]. Simultaneous inhibition of TGF-β and PD-L1 pathways can enhance overall therapeutic efficacy [57]. We compared the levels of TGF-β in tumor tissue and found that the levels were lower in the apatinib, MYOF knockdown, and combination groups compared to the control group (Supplementary Figure S4A). Immunohistochemistry also confirmed these findings (Supplementary Figure S4B). Additionally, an increase in TGF-β RNA expression levels was also observed in RKO and HCT116 cells (Supplementary Figure S4C). Immunohistochemistry also revealed significantly reduced PD-L1 expression in tumor tissues of the apatinib-treated, MYOF knockdown, and combination groups compared to controls (Supplementary Figure S5). In conclusion, our data suggest that apatinib treatment or MYOF knockdown represents a promising therapeutic approach for CRC, potentially enhancing anti-tumor immunity.

### 3.8. Synergistic Effects of Combined Treatment with Apatinib or Knocking Down MYOF and PD-1 Antibody

**Figure 8:**
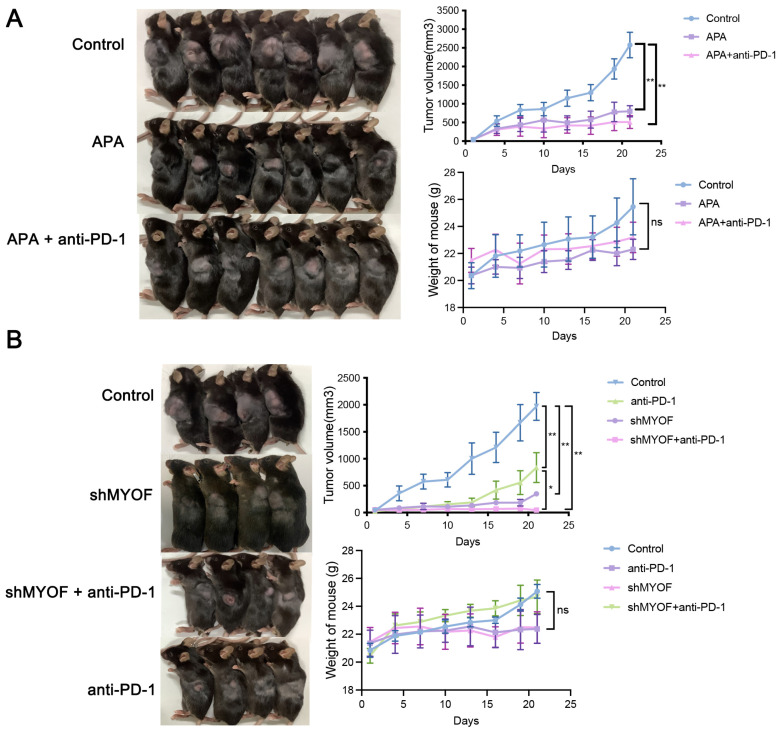
Apatinib or MYOF knockdown combined with Anti-PD-1 therapy further inhibits the progression of CRC in mice. (**A**) MC38 WT cells were subcutaneously implanted in mice. Tumor volume was recorded every 5 days. Representative images of C57BL/6 mice treated with apatinib and/or PD-1 antibody, growth curves of MC38 tumors, and body weight curves of the mice. (**B**) MC38 WT and MC38 MYOF knockdown cells were subcutaneously implanted in mice. Representative images of C57BL/6 mice treated with apatinib and/or MYOF knockdown, growth curves of MC38 tumors, and body weight curves of the mice (* *p* < 0.05; ** *p* < 0.01; ns, not significant).

Our results indicate that MYOF inhibition, either through gene knockout or apatinib treatment, significantly downregulates PD-L1 protein levels and affects various innate and adaptive immune signaling pathways. These pathways likely contribute to reshaping the TME to enhance anti-tumor immunity. Next, we tested the potential of further blocking the PD-L1/PD-1 axis to sensitize tumors to PD-1 therapy. Mice bearing MC38 tumors were treated with apatinib alone or a combination of apatinib and PD-1 antibody. Apatinib monotherapy and the combination of apatinib with α-PD-1 both significantly inhibited tumor growth, as expected. Although the combination therapy group demonstrated a trend toward further slowing tumor growth compared to the apatinib monotherapy group, this difference did not reach statistical significance. The relatively short treatment duration may have limited the detection of statistically significant differences between the groups (Con vs. APA, *p* < 0.0001; Con vs. APA + anti-PD-L1, *p* < 0.0001) (Figure 8A). Furthermore, mice were treated with MYOF knockdown, PD-1 antibody, or a combination of MYOF knockdown and PD-1 antibody. The combination therapy significantly reduced tumor growth compared to control mice and those receiving monotherapy of PD-1 antibody (Con vs. anti-PD-L1, *p* = 0.0054; Con vs. shMYOF, *p* = 0.0002; Con vs. shMYOF + anti-PD-L1, *p* < 0.0001; anti-PD-L1 vs. shMYOF + anti-PD-L1, *p* = 0.0388) (Figure 8B). The body weight of mice in the combination group was comparable to that of other groups. These data suggest that combining MYOF inhibition with PD-1 antibody therapy offers potential therapeutic benefits.

## 4. Discussion

In this study, we describe a MYOF-targeted strategy in the field of CRC immunotherapy. By using bioinformatics methods, we identified MYOF, which is highly expressed in CRC patients and is associated with the immune microenvironment. There is an interaction between VEGFR2 and MYOF, and the VEGFR2 inhibitor apatinib can downregulate MYOF via proteasomal degradation. The downregulation of MYOF expression promotes the ubiquitination of PD-L1. Our findings suggest that the VEGFR2–MYOF–PD-L1 signaling pathway plays a crucial role in regulating the efficacy of CRC immunotherapy. Screening for MYOF status may help predict the responsiveness to ICB, and the combined use of the VEGFR2 inhibitor apatinib may bring more clinical benefits to patients with advanced CRC.

Previous studies in endothelial cells have reported that MYOF silencing leads to membrane repair defects and blocks VEGF-mediated intracellular signaling pathways. A previous study suggests that MYOF might form a complex with VEGFR2 and dynamin-2 through its SH3 domain in HUVEC cells [53]. Subsequent studies by the same group demonstrated that MYOF silencing simultaneously downregulated VEGFR2 and Tie-2 expression in endothelial cells, significantly inhibiting downstream signaling and angiogenesis [58]. In studies on clear cell renal cell carcinoma (CCRCC), immunohistochemical data showed a high correlation between MYOF overexpression and low VEGFR2 expression. Furthermore, MYOF silencing in CCRCC cell lines resulted in transcriptional downregulation of VEGFR2 expression, although not statistically significant [59]. In immunohistochemical results from patients with non-small cell lung cancer (adenocarcinoma and squamous cell carcinoma), MYOF and VEGFR2 expression were positively correlated [30]. Thus, while the relationship between MYOF and VEGFR2 has been reported, the findings remain limited. Particularly in the field of cancer, the relationship and mechanisms between the two are contingent upon cancer types. Our study undertook a series of investigations into the relationship between MYOF and VEGFR2 in CRC. We found a significant positive correlation between MYOF and VEGFR2 expression in CRC patient tissues. In the mutual exclusivity analysis of three CRC sample cohorts, MYOF and VEGFR2 consistently showed significant co-occurrence trends. Immunoprecipitation experiments in the RKO cell line confirmed the binding between MYOF and VEGFR2, and overexpression of VEGFR2 resulted in synchronous increases in MYOF expression levels in both RKO and HCT116 cells. Similar to results in endothelial cell studies, we observed upregulation of VEGFR2 expression following MYOF overexpression. However, the proportion of MYOF upregulation after VEGFR2 overexpression was significantly higher. This may be related to the formation of a complex between VEGFR2 and MYOF, which mutually promotes protein stability, although the underlying complexity requires further experimental exploration.

Additionally, we provide evidence that apatinib exerts additional anti-tumor effects by inhibiting the activation of VEGFR2, thereby affecting the expression of MYOF. First, there is a positive correlation between the expression of MYOF and VEGFR2, and experiments have confirmed that MYOF and VEGFR2 interact, with apatinib inhibiting their binding. Second, apatinib promotes the degradation of MYOF through the ubiquitin–proteasome pathway, and this degradation is reversed after VEGFR2 overexpression. MYOF has become a new hotspot in tumor research. Previous studies on MYOF inhibitors mainly focused on synthesis, such as HJ445A and WJ460, but they have not yet been put into clinical use [60,61]. Here, we first demonstrate that apatinib can regulate MYOF expression, and its pharmacology and safety are well studied.

CRC can be categorized into two types based on mutation status: one type is tumors with dMMR–MSI-H markers, which have a generally high mutation burden (over 12 mutations per million DNA bases), and the other type is tumors with pMMR–MSS markers, which have a significantly lower mutation burden (fewer than 8.24 mutations per million DNA bases) [62]. In dMMR-MSI-H tumors, the MHC class I peptide complexes expressed on the surface of tumor cells include mutated peptides, which can be recognized by the immune system as neoantigens, thereby triggering and enhancing immune responses [63]. These tumors exhibit a high degree of immune cell infiltration, particularly with large numbers of CD8+ TILs, T helper 1 (TH1), CD4+ TILs, and macrophages [64,65]. Compared to patients with dMMR/MSI-H CRC, single-agent immunotherapy has not demonstrated significant clinical benefits in pMMR/MSS colorectal cancer patients, who constitute the majority of colorectal cancer cases. In the KEYNOTE-164 and CheckMate 142 studies, pMMR/MSS tumor patients did not exhibit a treatment response [4,11]. This could be attributed to insufficient immune cell recruitment in the tumor microenvironment, a key barrier affecting treatment efficacy. A promising strategy is to combine immunotherapy with other antitumor agents targeting different pathways to increase the immunogenicity of the tumor microenvironment, thereby converting “cold” tumors into “hot” tumors [66]. A prospective study found that the group treated with PD-1 inhibitors combined with apatinib had better progression-free survival (PFS) and overall survival (OS) than the monotherapy group, with higher objective response rates (ORR) and disease control rates (DCR) in the combination group [67]. The recent NEOCAP trial demonstrated that camrelizumab combined with apatinib as neoadjuvant therapy showed promising antitumor activity in patients with locally advanced mismatch repair-deficient or microsatellite instability-high colorectal cancer [68]. Our animal experiment results also proved that knockdown of MYOF or apatinib treatment increased the infiltration of CD8+ T cells, M1 macrophages, and activated DC cells, promoting anti-tumor immunity. The results of monotherapy with apatinib or combination therapy with apatinib and PD-1 antibodies showed a trend toward slowing tumor growth with the combination therapy, but statistical significance was not achieved. This may be due to the predominant antiangiogenic effects of apatinib compared to its anti-PD-1 effects or because the PD-1/PD-L1 pathway had already been modulated, thereby reducing the additional benefit of PD-1 antibodies. Furthermore, it was observed that MYOF inhibition combined with PD-1 antibodies exhibited a significant synergistic effect compared to PD-1 antibody monotherapy, further demonstrating that apatinib is not a single-target inhibitor. Notably, combination therapy strategies remain highly promising, as evidenced by previous studies exploring dual blockade approaches [69].

Our study emphasizes that MYOF is an inspiring target for converting cold tumors into hot tumors. Targeting MYOF alongside ICB therapy or using apatinib as an adjuvant treatment can enhance anti-tumor efficacy. However, our research has some limitations. First, due to the complexity of the regulatory mechanisms, we were unable to clearly explain the binding sites and activation mechanisms among VEGFR2, MYOF, and PD-L1. Second, although we observed that VEGFR2 overexpression significantly increased MYOF expression, apatinib may regulate MYOF through mechanisms other than VEGFR2. Lastly, the role of MYOF in clinical pMMR/MSS CRC patients requires further exploration.

## 5. Conclusions

Our study highlights the pivotal role of MYOF in regulating PD-L1 expression and reprogramming the colorectal cancer microenvironment. We demonstrate that apatinib downregulates MYOF via VEGFR2-mediated proteasomal degradation, thereby promoting PD-L1 degradation and enhancing the immune microenvironment. These effects, coupled with the observed synergistic efficacy of apatinib and PD-1 antibodies in inhibiting colorectal cancer growth, underscore the translational potential of targeting the VEGFR2–MYOF–PD-L1 axis. This research not only advances our understanding of tumor immunology but also paves the way for innovative therapeutic strategies to improve outcomes for colorectal cancer patients.

## Data Availability

The datasets sharing used and/or analyzed during the current study are available from the corresponding author Tianhui Hu on reasonable request.

## Supplementary Materials

The following supporting information can be downloaded at: https://www.mdpi.com/article/10.3390/cancers17030524/s1, Figure S1: Transcriptome sequencing of normal and cancerous intestinal cells; Figure S2: Effect of MYOF knockdown and apatinib treatment on PD-L1 mRNA expression in RKO cells; Figure S3: FACS analysis of CD8+ and CD4+ T lymphocytes infiltrating mouse spleen; Figure S4: The effect of apatinib and MYOF knockdown on transforming growth factor-β; Figure S5: Immunohistochemical staining and analysis of PD-L1 expression in tumor tissues; File S1: The original Western blot figures.
