# Supplementary material for: Apatinib Degrades PD-L1 and Reconstitutes Colon Cancer Microenvironment via the Regulation of Myoferlin
Source: Cancers (Basel). 2025 Feb 5;17(3):524. doi: 10.3390/cancers17030524 (PMC11816266; doi:10.3390/cancers17030524)

Uncropped gels for Western Blots

Figure 2B

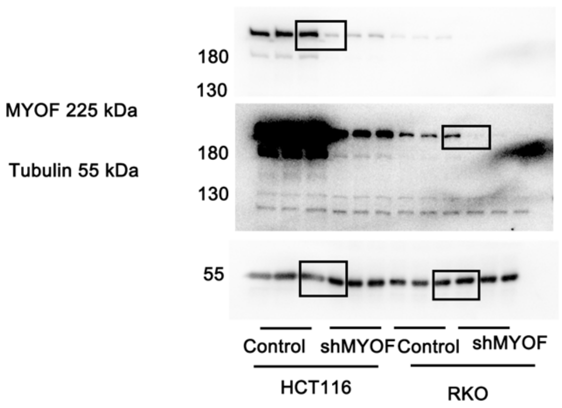

additional information:

Different exposure times on the same Western blot membrane

Figure 3D

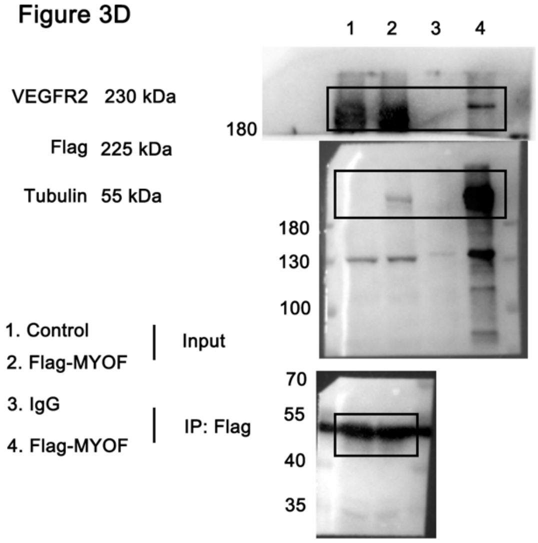

Figure 4C

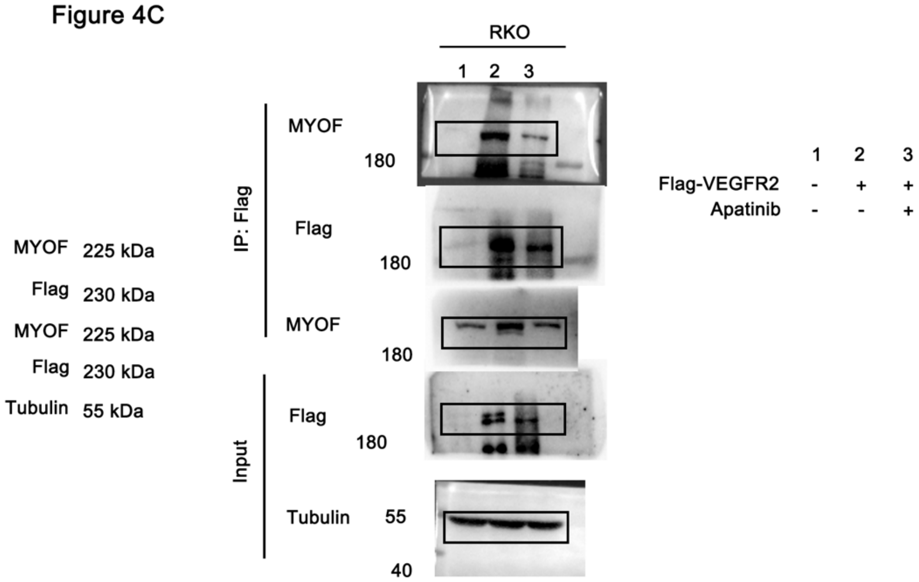

Figure 3E

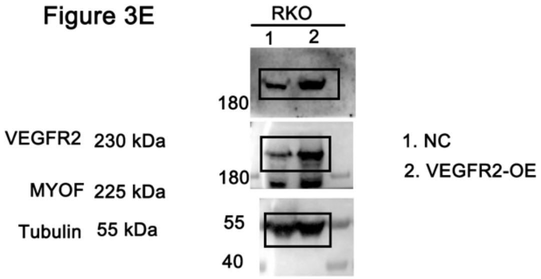

Figure 4B

VEGFR2 230 kDa

MYOF 225 kDa

Tubulin 55 kDa

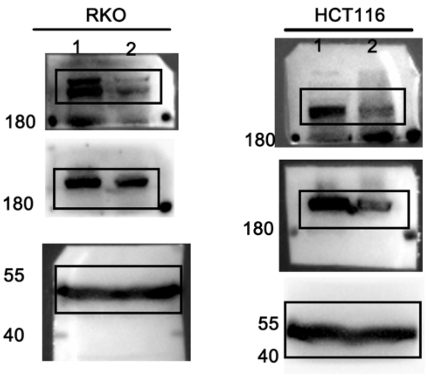

1. PBS

2. Apatinib

Figure 4D

MYOF 225 kDa

Tubulin 55 kDa

	1	2	3	4	5	6	7	8	9	10	11	12
MG132 (20 μM)	-	-	-	-	-	-	-	-	+	+	+	+
Apa (10 μM)	-	-	-	-	+	+	+	+	+	+	+	+
CHX (50 μM)	+	+	+	+	+	+	+	+	+	+	+	+
Time (h)	0	2	4	8	0	2	4	8	0	2	4	8

Repeat 1 (HCT116)

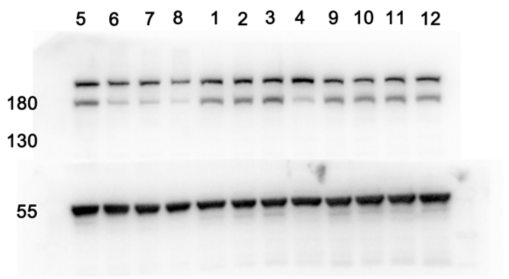

Repeat 1 (RKO)

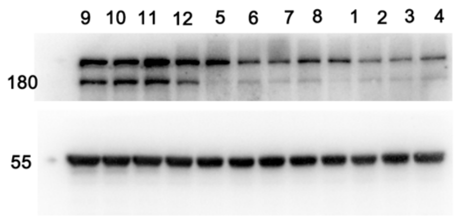

Repeat 2 (HCT116)

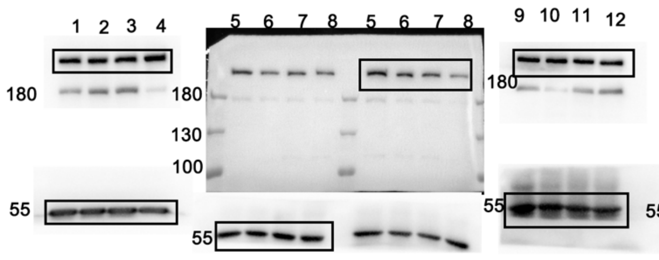

Repeat 2 (RKO)

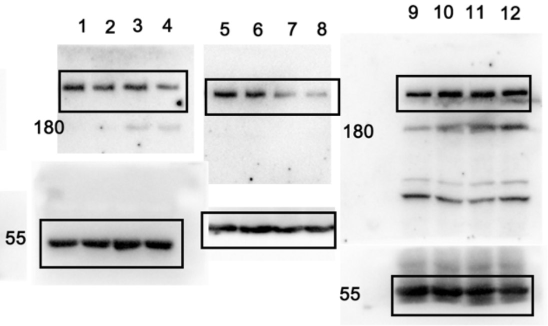

Repeat 3 (HCT116)

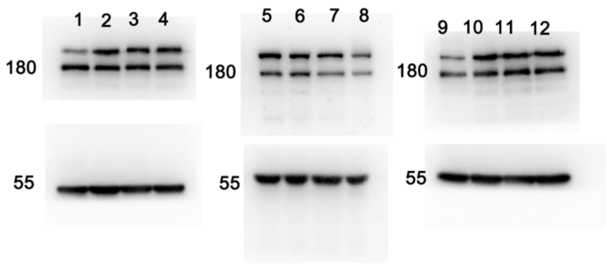

Repeat 2 (RKO)

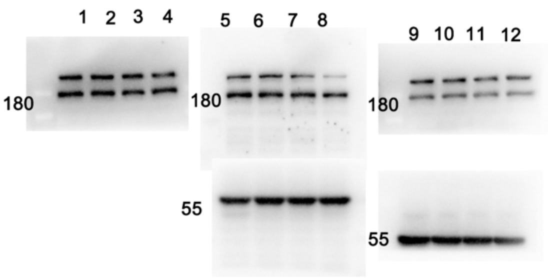

Figure 4F

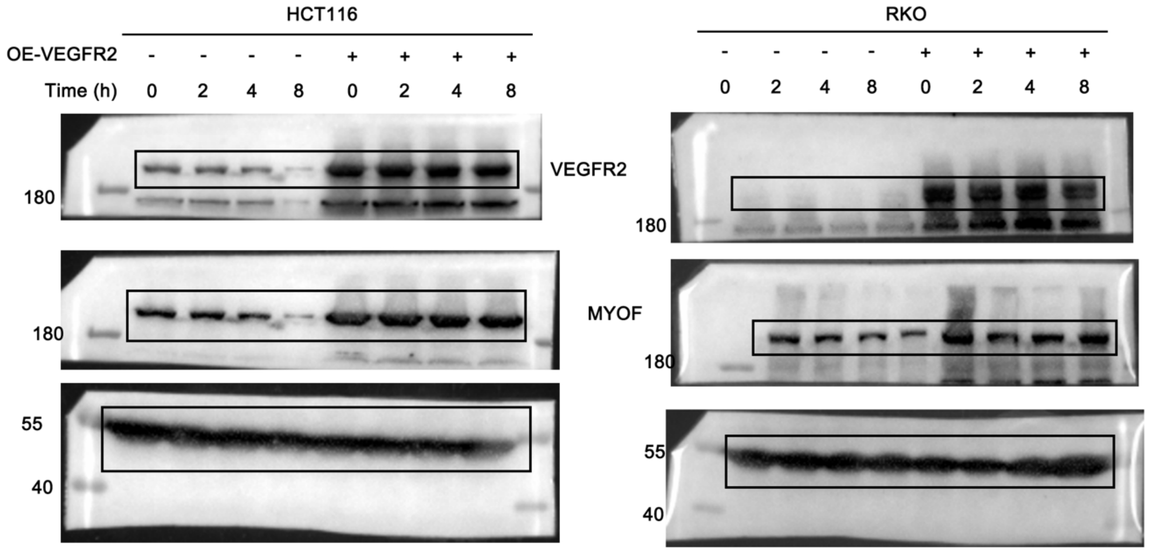

Figure 6A

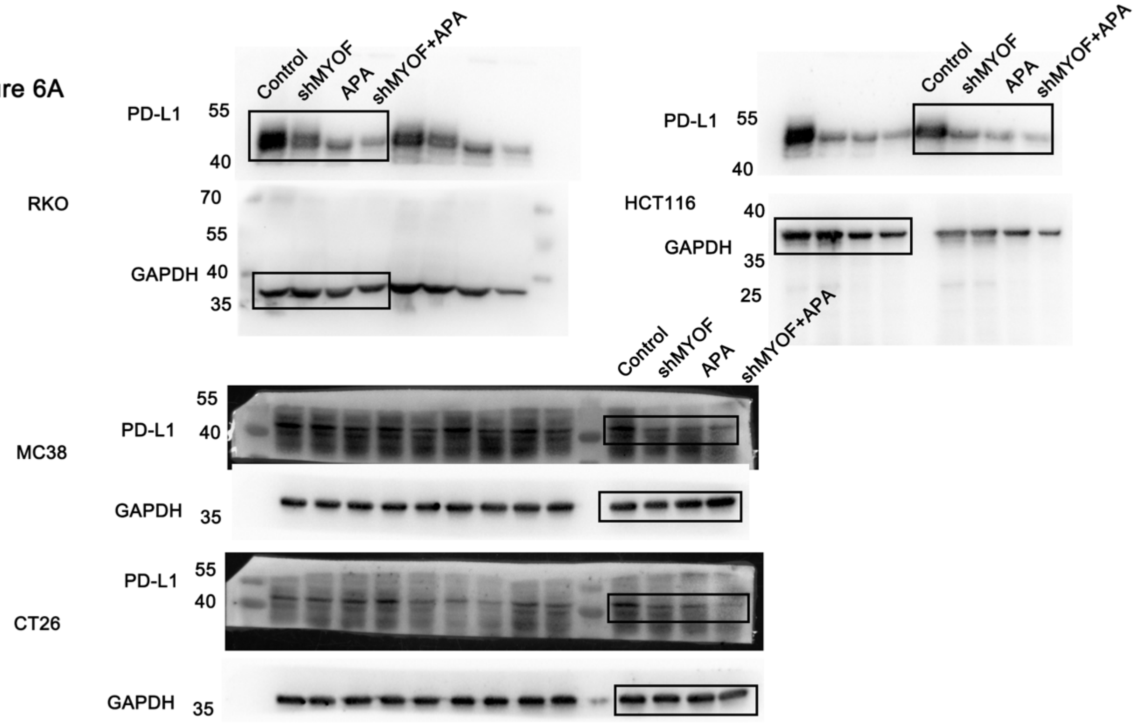

Figure 6B

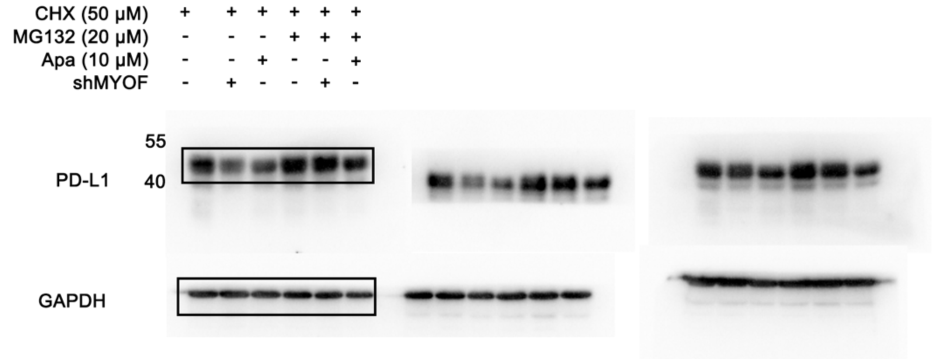

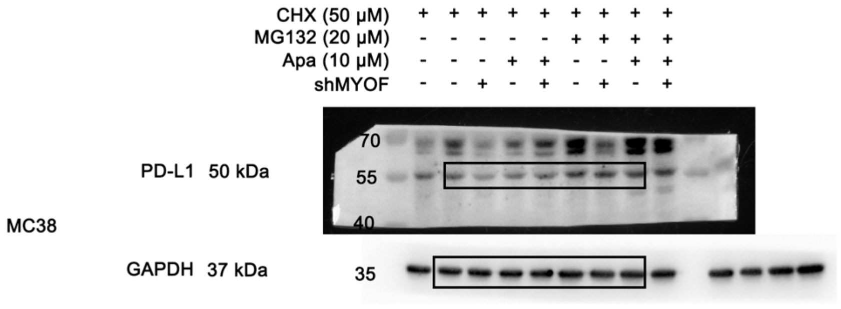

Figure 6C

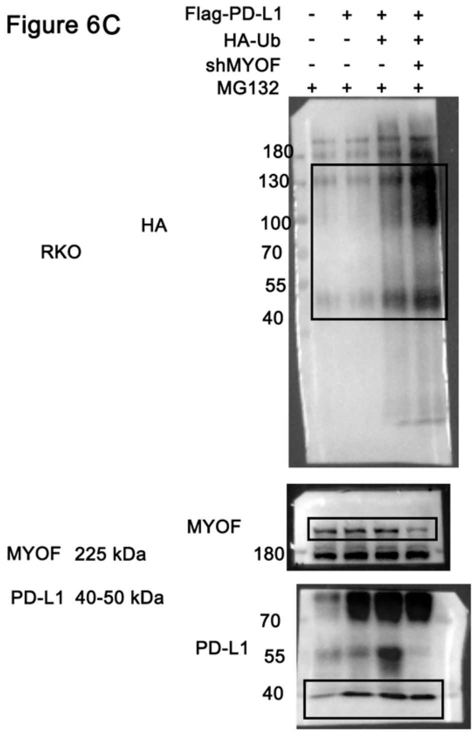

Figure 6D

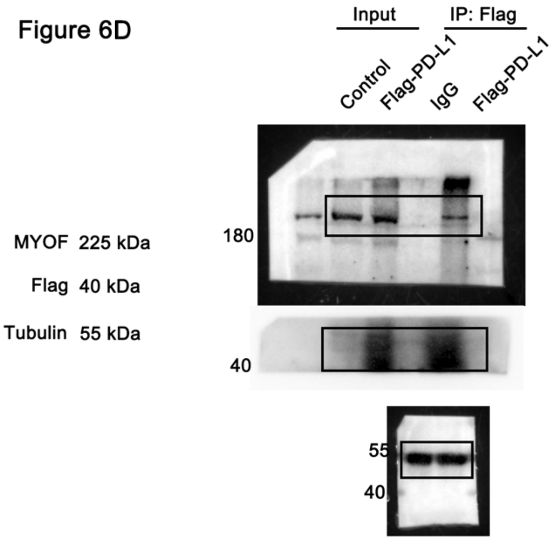

Figure 6E

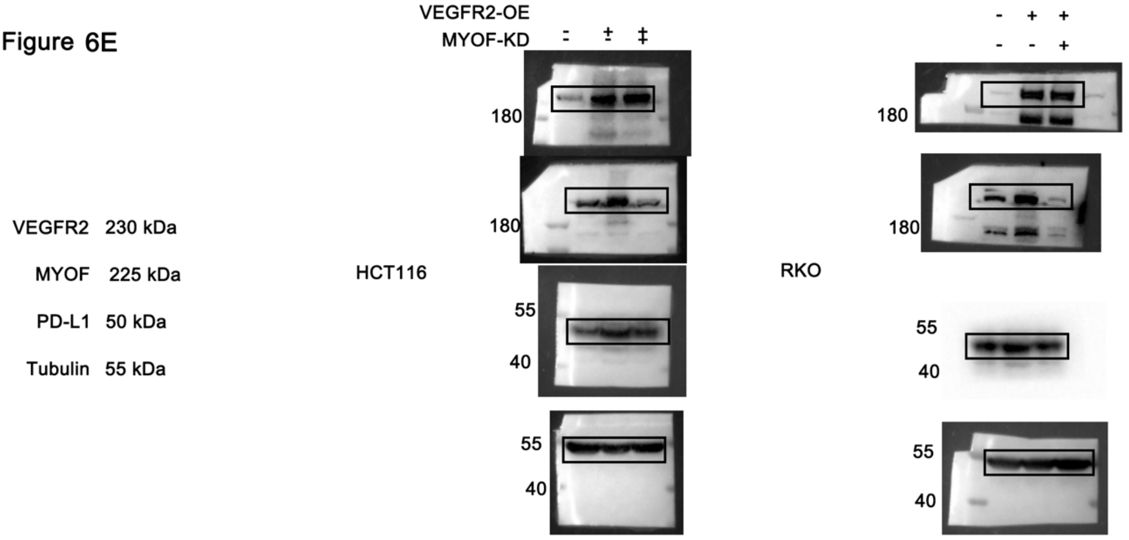

Supplement: Supplementary file 1 [file cancers-17-00524-s001.zip › cancers-3382271-File S1.pdf]
