# Supplementary material for: Apatinib Degrades PD-L1 and Reconstitutes Colon Cancer Microenvironment via the Regulation of Myoferlin
Source: Cancers (Basel). 2025 Feb 5;17(3):524. doi: 10.3390/cancers17030524 (PMC11816266; doi:10.3390/cancers17030524)
Supplement: Supplementary file 1 [file cancers-17-00524-s001.zip › cancers-3382271-Supplementary Figures.pdf]

Supplementary Information for

Apatinib degrades PD-L1 and reconstitutes colon cancer microenvironment via the regulation of Myoferlin

Chunyi Gao, Lu Chen, Lingying Zhao, Yongcheng Su, Miaomiao Ma, Wenqing Zhang, Xiaoting Hong, Li Xiao, Beibei Xu, and Tianhui Hu

This file includes:

Supplementary Figures S1 - S5

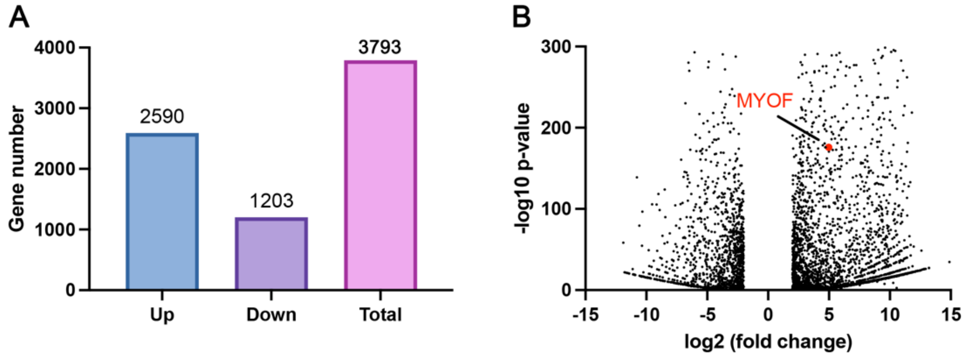

Figure S1: Transcriptome sequencing of normal and cancerous intestinal cells. (A)The number of upregulated and downregulated genes in the comparison between RKO and HIEC cells. (B) The distribution of MYOF in the volcano plot generated from the transcriptome sequencing data of the cell lines.

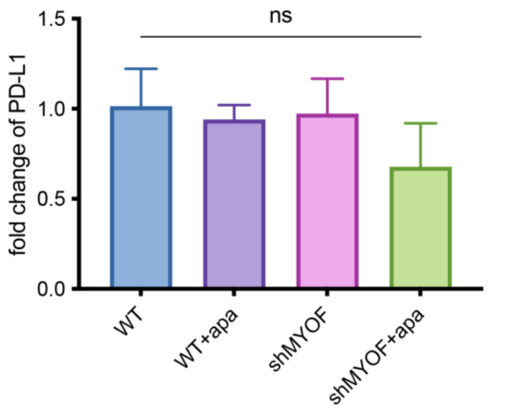

Figure S2: Effect of MYOF knockdown and apatinib treatment on PD-L1 mRNA expression in RKO cells.

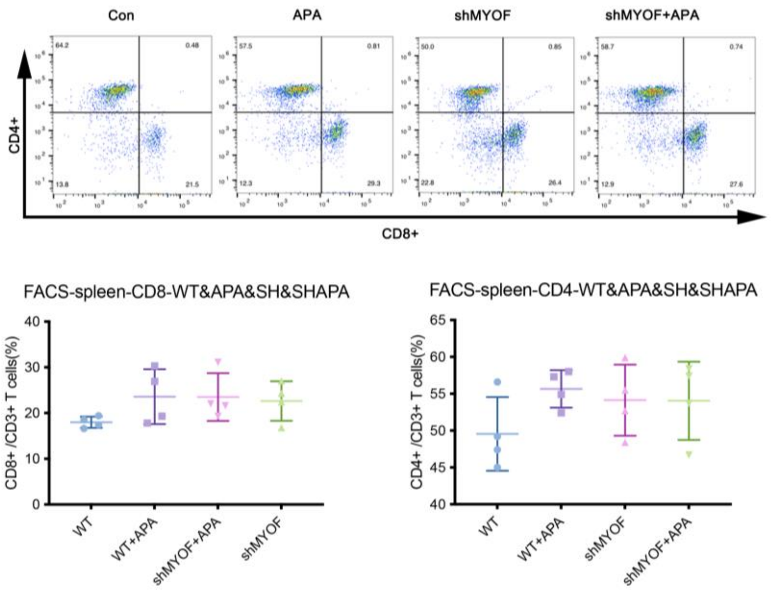

Figure S3: FACS analysis of CD8+ and CD4+ T lymphocytes infiltrating mouse spleen.

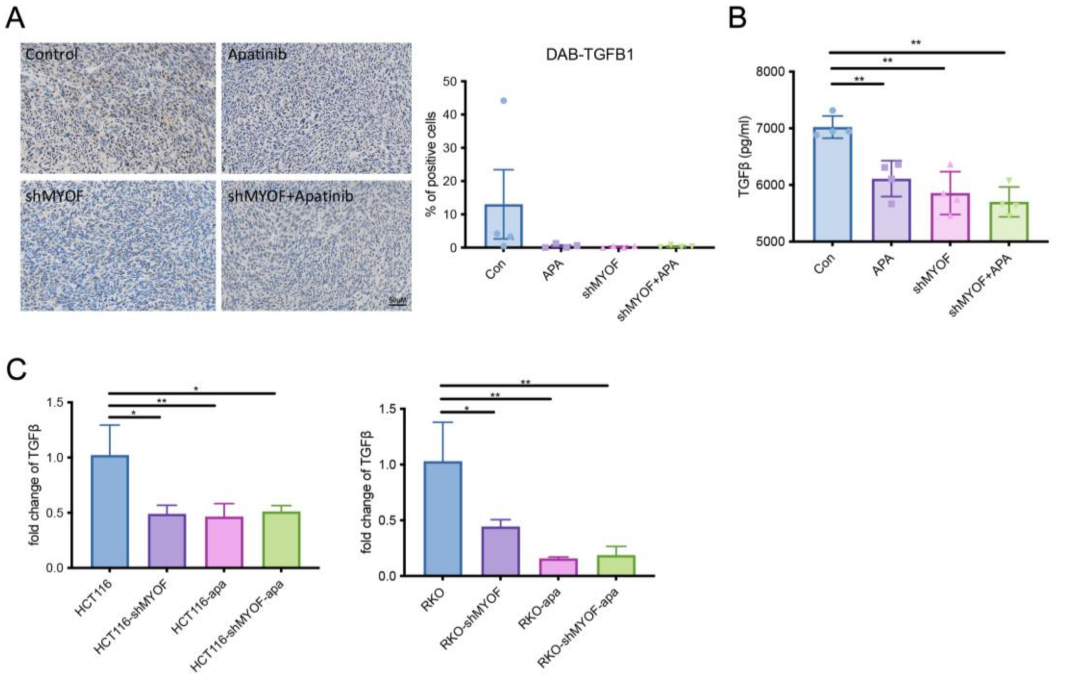

Figure S4: The effect of apatinib and MYOF knockdown on transforming growth factor-β. (A) Immunohistochemical staining and analysis of TGF-β expression in tumor tissues. (B) ELISA analysis of TGF-β levels in tumor tissue homogenates. (C) qPCR analysis of TGF-β mRNA expression in RKO and HCT116 cells. * $p < 0.05$; ** $p < 0.01$.

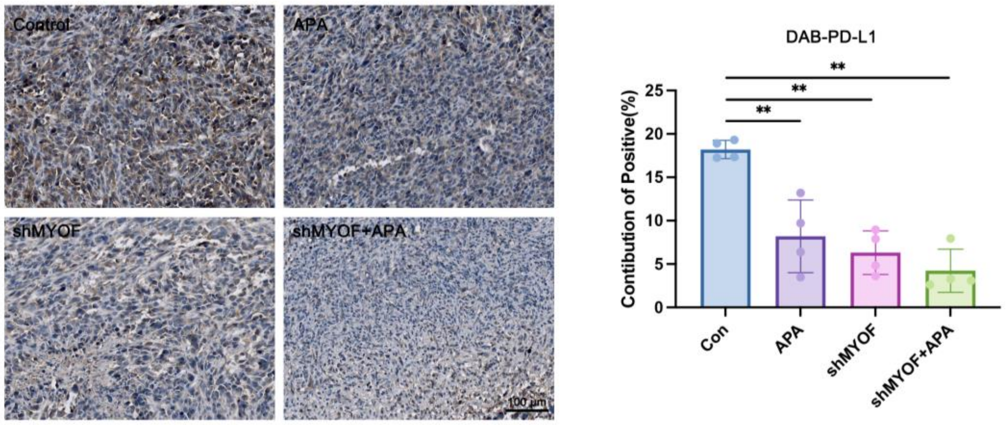

Figure S5: Immunohistochemical staining and analysis of PD-L1 expression in tumor tissues. ** $p < 0.01$.
